# How to measure monetary losses in gambling disorder? An evidence-based refinement

**DOI:** 10.1016/j.psychres.2017.12.004

**Published:** 2018-05

**Authors:** Gustavo C. Medeiros, Sarah A. Redden, Samuel R. Chamberlain, Jon E. Grant

**Affiliations:** a Department of Psychiatry, University of Texas Southwestern Medical Center, 5323 Harry Hines Blvd., Dallas 75390-9070, TX, USA; b Department of Psychiatry & Behavioral Neuroscience, University of Chicago, Chicago, IL, USA; c Department of Psychiatry, University of Cambridge, Cambridge, United Kingdom

**Keywords:** Addictions, Clinical Measures, Outcome Assessment

## Abstract

Diverse monetary measures have been utilized across different studies in gambling disorder (GD). However, there are limited evidence-based proposals regarding the best way to assess financial losses. We investigated how different variables of monetary losses correlate with validated assessments of gambling severity and overall functioning in a large sample of subjects with GD (n = 436). We found that relative monetary variables (i.e. when financial losses were evaluated in relation to personal income) showed the most robust correlations with gambling severity and overall psychosocial functioning. Percentage of monthly income lost from gambling was the variable with the best performance.

## Highlights

• Address an important gap: how to assess financial losses in gambling disorder?
• Evidence-based proposal using golden standard measures.
• The best monetary variables assess financial losses in relation to personal income.
• Percentage of monthly income lost from gambling was the best variable.
• Our findings have significant impact on clinical practice and research on GD.

## 1 Introduction

Gambling disorder (GD) and money are two almost inseparable concepts. Monetary losses are usually evaluated as a measure/proxy of gambling severity in clinical practice and research on GD (see for example Hodgins et al., 2004; Gee et al., 2005; Floyd et al., 2006; Petry et al., 2006; Weinstock et al., 2007; Grant et al., 2008; Diskin and Hodgins, 2009; Slutske et al., 2010; Medeiros et al., 2016; de Brito et al., 2016). Diverse monetary measures have been used across different studies. However, there is no clear consensus on the financial measure that best captures gambling severity and problems in psychosocial functioning. For example, the following monetary variables have been already reported in literature: 1) money spent on gambling per month/year (Hodgins et al., 2004, Diskin and Hodgins, 2009, de Brito et al., 2016); 2) percentage of income spent on gambling per month/year (Weinstock et al., 2007, Grant et al., 2008, Slutske et al., 2010, Medeiros et al., 2016); 3) money spent per gambling episode or per day of gambling (Gee et al., 2005, Floyd et al., 2006, Petry et al., 2006). Nonetheless, there are limited evidence-based proposals regarding the most valid financial measure to assess gambling severity and psychosocial functioning.

The financial variables that have been used in GD clinical practice and research can be classified into two categories: absolute and relative measures. Examples of two commonly used absolute measures are 1) money lost in gambling during a specific time range – usually losses per month (see Hodgins et al., 2004; Diskin and Hodgins, 2009; de Brito et al., 2016), and 2) money lost per gambling episode (see Gee et al., 2005; Floyd et al., 2006). Absolute financial variables do not compare the losses to the subjects’ income. Relative measures, on the other hand, evaluate financial loss in relation to personal income (see Weinstock et al., 2007; Grant et al., 2008; Slutske et al., 2010; Medeiros et al., 2016). A consensus of specialists suggested that “the critical aspect may not be the absolute quantity of money lost but the proportion of total income or personal expendable income that is lost” (Walker et al., 2006). However, no study has compared different monetary measures in a systematic way. In this context, the use of diverse assessments of monetary losses in GD may be in part a result of an absence of evidence-based suggestions.

In light of this discussion, we investigated how different measurements of monetary loss correlated with validated assessments of gambling severity and of overall psychosocial functioning. Our hypothesis was that relative monetary measures, instead of absolute monetary variables, would show stronger correlations with measures of gambling severity and overall psychosocial functioning. More specifically, based in clinical experience and specialists’ suggestions, we postulated that percentage of monthly income lost due to gambling would constitute the superior financial measure in GD.

## 2 Methods

### 2.1 Sample and recruitment

We evaluated 436 individuals that participated in clinical trials on pharmacotherapy or psychotherapy in GD. The mean and median age were, respectively, 47.3 (± 11.3) and 48.0; 44.7% (n = 195) were males. The current study used the combined database from seven published studies: Kim et al. (2001, 2002), Grant and Potenza (2006), Grant et al. (2007, 2009, Grant et al., 2010a, Grant et al., 2010b 2013). Additionally, we included a subset of subjects (those recruited at the University of Minnesota and at the University of Chicago) from three multicentric studies: Grant et al. (2003, 2006, Grant et al., 2010a, Grant et al., 2010b. All participants met the GD criteria according to the Diagnostic and Statistical Manual of Mental Disorder 5 – DSM 5 (American Psychiatric Association [APA], 2013) (subjects recruited before 2013 were retrospectively examined using the DSM-5). Data at baseline (first visit) were used for the current study. The sample was enlisted in the metropolitan areas of Chicago, IL and Minneapolis, MN through advertisements on the internet, public places and newspapers. Participants were compensated with a US$ 50 gift card to local department stores.

### 2.2 Measures

#### 2.2.1 Demographics

Subjects were assessed for age, gender, marital status, educational level and ethnicity.

#### 2.2.2 Monetary variables

We evaluated 3 primary measures: 1) money lost last year in gambling (in US$). Money lost (expenditure) was defined as the total amount of money spent on gambling and was assessed in terms of net expenditure i.e. [money available at the beginning of the session] plus [subsequent withdrawals or borrowing] less [money available and the end of the session]. This approach to monetary losses in GD is arguably considered the most appropriate (Walker et al., 2006); 2) personal annual income last year in US$ (including income from formal work and additional earning such as investments); and 3) gambling frequency in gambling episodes per week. A gambling episode was defined as a session of bets without significant interruption by other activities such as: a) work; b) going home; c) interactions with others not associated with gambling; d) other activities. For example, three separated visits to the casino over the course of one day would count as 3 gambling episodes.

Using these values, we calculated the following final absolute and relative (percentage of income) monetary variables. The variables computed were: a) average monthly monetary losses in gambling (money lost last year in gambling/12); b) average money lost per gambling episode (money lost last year/[gambling frequency in times per week × 52.2]). Note that 52.2 is the average number of weeks in a year; c) average percentage of monthly income lost in gambling ([money lost last year/12]/[income last year/12]); and d) percentage of income lost per gambling episode ({money lost last year/[gambling frequency in times per week × 52.2]}/income last year).

#### 2.2.3 Primary form of gambling

Assessed with a semi-structured questionnaire in which each subject reported the primary form of gambling (one or two main forms).

#### 2.2.4 Gambling severity

Investigated by the Gambling Symptom Assessment Scale (GSAS) and the Yale-Brown Obsessive-Compulsive Scale Modified for Pathological Gambling (PG-YBOCS). The GSAS is a 12-item self-report questionnaire that investigates gambling severity (Kim et al., 2009). The items assess urges; gambling involvement (time, frequency, duration, control); anticipatory excitement/tension; pleasure in gambling; emotional and personal problems due to gambling behavior. The participant has 5 answer choices for each question 0–4), therefore, the final score ranges between 0 and 48 (Kim et al., 2009). GSAS showed good internal consistency (Cronbach's alpha = 0.869) and test-retest reliability (rho = 0.56; p = 0.047). The scale showed a good validity as well (Kim et al., 2009).

The PG-YBOCS is a 10-item clinician-administered of gambling severity developed by Pallanti et al. (2005). Five questions assess gambling thoughts/urges while the other 5 items examine gambling-related behavior (Pallanti et al., 2005). PG-YBOCS showed an excellent internal consistency (Cronbach's alpha = 0.970) and an intraclass correlation coefficient of 0.970. With regards to the validity, PG-YBOCS demonstrated a very strong correlation (*r* = 0.895; p < 0.001) with a previous scale of gambling severity – the South Oaks Gambling Screen [SOGS] (Pallanti et al., 2005).

#### 2.2.5 Overall Functioning

This study assessed three main measures of overall functioning: 1) clinical global impression [Clinical Global Impression Scale – CGI]; 2) functional impairment [Sheehan Disability Scale]; and 3) quality of life [Quality of Life Inventory – QOLI].

The CGI is a standardized clinician-administered instrument that assesses the global severity of an illness. This scale was originally designed by Guy (1976) and has been widely used in mental health research. The scale has demonstrated strong psychometrics in different populations (see for example Khan et al., 2002; Kadouri et al., 2007; Hedges et al., 2009).

The Sheehan Disability Scale consists of three self-rated questions that investigates how much the individual´s life has been affected/impaired by the mental disorder (Leon et al., 1997). The items examine disability in different domains: a) work; b) social life/leisure activities and c) home/family life. The sum of the three domains provides an overall score, which was used in our study. In a study with 1001 individuals, the Sheehan Disability Scale exhibited good reliability (Cronbach's alpha = 0.89 and inter-item correlation between 0.70 and 0.79) and substantial validity (80% of the subjects with a mental disorder showed elevated scores in this scale) (Leon et al., 1997).

The QOLI is a 17-item self-administered scale that examines the person's quality of life in different areas (Mendlowicz and Stein, 2000). The weighted sum of the questions provides an overall score. This scale showed Cronbach's alphas ranging from 0.77 and 0.89, and test re-test correlations between 0.80 and 0.91 (Mendlowicz and Stein, 2000).

### 2.3 Statistical analysis

First, we analyzed the distribution of the monetary variables using One-Sample Kolmogorov-Smirnov tests. As all financial variables demonstrated a non-parametric dispersal, we investigated the correlations between the monetary variables and gambling severity/overall psychosocial functioning using Spearman's Correlation Coefficients (*rho*). Size of correlation was categorized according to Cohen's classification for behavioral sciences – see footnotes in Table 1 for details.

**Table 1:** Description of monetary measures in subjects with gambling disorder^a^ (total n = 436).

Monetary variables	Mean (Standard deviation)	Median	Range	Skewness (Standard error)	Kurtosis (Standard error)
− Annual Income^a^ (in US$)	28,794.19 (32,883.99)	25,000.00	250,000.00	2.29 (0.12)	8.81 (0.23)
− Monthly Monetary Losses from Gambling^b^ (in US$)	1285.65 (1817.33)	666.67	15,833.33	2.93 (0.12)	12.91 (0.23)
− Money Lost per Gambling Episode [N^c^ = 254] (in US$)	53.47 (104.45)	18.31	895.03	4.45 (0.15)	25.46 (0.30)
− Percentage of Monthly Income Lost from Gambling [N = 294]	66.11 (130.24)	40.00	1500.00	7.79 (0.14)	74.03 (0.28)
− Percentage of Income Lost per Gambling Episode [N = 184]	2.25 (4.29)	0.89	27.62	3.60 (0.18)	13.54 (0.36)

a One hundred forty-two (32,6%) subjects did not have any kind of personal income. They were included in the calculations of annual income.

b All monetary variables refer to net expenditure i.e. [money available at the beginning of the session] plus [subsequent withdrawals or borrowing] less [money available and the end of the session] (Walker et al., 2006).

c N = Number of valid subjects for the variable. If the *N* is not displayed, the total sample (n = 436) was evaluated for the variable.

### 2.4 Ethics

Data collection for this study was approved by Institutional Review Boards for the University of Chicago and University of Minnesota. All subjects provided written informed consent.

## 3 Results

The mean and median number of DSM-5 criteria fulfilled by the studied sample were, respectively, 7.2 (± 1.3) and 7.0. The mean/median scores for the GSAS were, respectively, 35.3 (± 13.0) and 34.0. The same data for the PG-YBOCS were 20.8 (± 5.1) and 20.0. In light of these scores, the participants of the current study would be classified as *moderate* according to the DSM-5 (APA, 2013) and as *severe* according to the GSAS (Kim et al., 2009) and The fact that we investigated subjects mainly in the moderate to severe range may explain the high percentage of income (median 40.0%) spent on gambling in our sample. Table 1 reports the main monetary data on our sample.

In terms of the gambling severity scales, two hundred forty-six subjects answered both the GSAS and the PG-YBOCS; one hundred eighteen participants completed the GSAS and twenty-seven answered only the PG-YBOCS. The *rho* between GSAS and PG-YBOCS was 0.616 (p < 0.001; n = 246), which is considered large by Cohen's (1988) standards. Concerning gambling frequency, the mean and median number of gambling episodes per week were, respectively, 6.75 (± 10.3) and 2.50. With respect to the primary games played by the studied sample, the most frequent forms of gambling were: 1) electronic gaming machines [slots machines, keno, video bingo] (n = 363; 83.3%); 2) card games [poker, blackjack, other card games] (n = 131; 20.2%); 3) lottery (n = 31; 7.1%); 4) pull tabs/scratch cards (n = 30; 6.9%); 5) Video Poker (n = 18; 4,1%); and 6) Bingo (n = 18; 4.1%).

Monthly monetary losses from gambling and money lost per gambling episode (absolute monetary variables) resulted in correlation coefficients in the *not relevant* to *small* range in relation to variables of gambling severity and overall functioning (see Table 2). The average effect size for these two variables were 0.013 (± 0.016) and 0.011 (± 0.009), respectively. Percentage of monthly income lost from gambling and percentage of income lost per gambling episode (relative monetary variables) tended to show more robust correlation coefficients when compared to the absolute monetary losses. The average effect size for these two variables were, respectively, 0.096 (± 0.076) and 0.074 (± 0.112).

**Table 2:** Size of correlation^a^ between monetary variables, gambling disorder severity and overall functioning (total n = 436).

Monetary variables		Gambling disorder severity		Overall functioning		
		G-SAS^b^	PG-YBOCS^b^	Clinical global impression^b^	Functional impairment^b^	Quality of life^b^
Absolute values	Monthly Monetary Losses from Gambling^c^	*SMALL*	*SMALL*	*NOT RELEVANT*	*NOT RELEVANT*	*NOT RELEVANT*
		*r*^d^ = 0.205	*r* = 0.108	*r* = 0.040	*r* = 0.067	*r* = − 0.068
		*r*^*2*d^ = 0.042	*r*^*2*^ = 0.012	*r*^*2*^ = 0.002	*r*^*2*^ = 0.004	*r*^*2*^ = 0.005
		sig^g^ < 0.001	sig = 0.075	sig = 0.533	sig = 0.533	sig = 0.467
		N^g^ = 364	N = 273	N = 250	N = 223	N = 115
	Money Lost per Gambling Episode^c^	*NOT RELEVANT*	*SMALL*	*NOT RELEVANT*	*SMALL*	*NOT RELEVANT*
		*r* = 0.066	*r* = − 0.119	*r* = 0.091	*r* = − 0.159	*r* = − 0.072
		*r*^*2*^ = 0.004	*r*^*2*^ = 0.014	*r*^*2*^ = 0.008	*r*^*2*^ = 0.025	*r*^*2*^ = 0.005
		sig = 0.337	sig = 0.223	sig = 0.286	sig = 0.152	sig = 0.554
		N = 213	N = 102	N = 141	N = 83	N = 69
						
Relative values	Percentage of Monthly Income Lost from Gambling^c^	*SMALL*	*MEDIUM*	*SMALL*	MEDIUM	*MEDIUM*
		*r* = 0.141	*r* = 0.432	*r* = 0.120	*r =* 0.352	*r* = − 0.369
		*r*^*2*^ = 0.020	*r*^*2*^ = 0.187	*r*^*2*^ = 0.014	*r*^*2*^ = 0.124	*r*^*2*^ = 0.136
		sig = 0.035	sig < 0.001	sig = 0.079	sig < 0.001	sig = 0.001
		N = 224	N = 135	N = 217	N = 103	N = 73
	Percentage of Income Lost per Gambling Episode^c^	*SMALL*	*NOT RELEVANT*	*SMALL*	*SMALL*	LARGE
		*r* = − 0.239	*r* = 0.039	*r* = 0.125	*r* = − 0.157	*r* = − 0.522
		*r*^*2*^ = 0.057	*r*^*2*^ = 0.002	*r*^*2*^ = 0.016	*r*^*2*^ = 0.025	*r*^*2*^ = 0.272
		sig = 0.004	sig = 0.827	sig = 0.187	sig = 0.425	sig = 0.004
		N = 143	N = 34	N = 113	N = 28	N = 28

a Size of correlation: *NOT RELEVANT* if absolute correlation coefficient ≥ 0.000 and < 0.100; *SMALL* if absolute correlation coefficient ≥ 0.100 and <0.300; *MEDIUM* if absolute correlation coefficient ≥ 0.300 and < 0.500; *LARGE* if absolute correlation coefficient ≥ 0.500 (Cohen, 1988).

b G-SAS = the Gambling Symptom Assessment Scale (Kim et al., 2009); PG-YBOCS = the Pathological Gambling Adaptation of the Yale-Brown Obsessive-Compulsive Scale (Pallanti et al., 2005). Clinical Global Impression = Clinical Global Impression Scale (Guy, 1976); Functional Impairment = Sheehan Disability Scale. Quality of Life = Quality of Life Inventory (Frisch, 1994).

c All monetary variables are reported in American Dollars and refer to net expenditure i.e. [money available at the beginning of the session] plus [subsequent withdrawals or borrowing] less [money available and the end of the session] (Walker et al., 2006).

d *r*: spearman's correlation coefficient; *r*^*2*^*=* effect size; sig: level of significance for correlation coefficient; N: number of subjects evaluated for the specific correlation.

## 4 Discussion

This study used a large sample of individuals with GD to address an important issue in GD: what is the most clinically relevant method to assess financial losses? Our results suggest that percentage of monthly income lost in gambling may be the most clinically valid measurement of financial losses.

This study observed that relative monetary variables (when compared to absolute monetary measures) showed the strongest correlations with gambling severity and overall functioning. Absolute measures do not take into account the subject's income. However, the same monetary loss may have little or a highly significant impact on the disordered gambler depending on the available financial resources. As a result, absolute financial measures (when compared to relative monetary variables) have limited usefulness in terms of gambling severity and, particularly, of overall psychosocial functioning.

Measures that assessed monthly monetary losses better correlated with the comparison scales, specifically in terms of GD severity, than variables that investigated losses per gambling episode. GD is characterized by “persistent and recurrent problematic behavior” according to the Diagnostic and Statistical Manual of Mental Disorder 5. Therefore, repetitive losses during a certain period may better simulate GD than fewer episodes of significant losses. In this scenario, Walker et al. (2006) further suggest that the monthly monetary losses should be an average of longer periods in order to comprehend the chronicity of the disorder. In this research, we used the average of the financial losses in the last year.

### 4.1 Limitations

This study should be interpreted in light of its limitations. Previous research has suggested problems in retrospective self-reported assessments of monetary expenditure in gambling (Blaszczynski et al., 1997, Blaszczynski et al., 2006, Wood and Williams, 2007). These financial estimations may be affected by factors such recall bias, sense of desirability of the behavior and interpretation of the questions (Wood and Williams, 2007). In this context, it is possible that the values reported by the studied sample may have some degree of inaccuracy. One fact that may had minimized some of these issues in this research was the use of a standardized semi-structured questionnaire applied by trained researchers. This may have partially mitigated the problems in interpretation since the previously instructed interviewers explained clearly the questions and, if needed. provided further explanations. Moreover, the assessments used in our study closely resemble the reality in clinical practice and research.

Another limitation was the use of data pooled from multiple trials, which in some cases had differing study designs. However, we focused on baseline data, so individual study differences were unlikely to affect the overall results. Furthermore, some characteristics of the studies may have further alleviated possible biases originated from the polled data. First, all interviewers received the same training regarding the application of the semi-structured questionnaire. Second, the multiple trials grouped in this study were conducted by the same clinical research laboratory (i.e. changes in the investigation team were minimal). This provides additional consistency in the application of the research procedures. Moreover, all trials used the same questions to extract data on financial losses, annual income and gambling frequency. These features may assure a good level of reliability for the current research.

In addition, our sample consisted of subjects who mainly participated in pharmacological studies. Therefore, caution is needed when extrapolating our results to broader groups of disordered gamblers. For example, a meta-analysis suggested that younger individuals may prefer psychological treatments (McHugh et al., 2013) [note that the mean age in our study was 47.3 (± 11.3) years]. Finally, it is important to mention that we performed correlation analyses to study the relationship between absolute numbers and percentages. Correlations are, in theory, designed to analyze two absolute numbers. Therefore, the utilization of percentages (relative measures) in these analyses may lead to less reliable results. Despite the limitations, our study provides evidence-based data that adds to our current understanding regarding the most appropriate way to measure monetary losses in GD.

### 4.2 Future studies

The current study evaluated monetary losses due to gambling in general (i.e. no distinction was made between the main form of gambling and other secondary types of games). Future studies may address if this distinction may lead to better monetary measures. Some of the individuals with GD do not have a formal income. It is possible that expendable money available (i.e. loans, help from relatives and friends, social security, other financial sources) may be used in these cases. Future research should evaluate this form of measurement. Finally, longitudinal studies that assess money lost in alternative ways are needed. Examination through prospective personal diaries (Wood and Williams, 2007) and instantaneous registration of values thought the venue may decrease possible inaccuracies in the retrospective report of financial losses.

### 4.3 Conclusions

Our study suggests that relative monetary variables (i.e. when financial losses were evaluated in relation to personal income) - instead of absolute monetary measures – shows the most consistent and robust correlations with gambling severity and overall psychosocial functioning. Among the relative financial measures, percentage of monthly income lost from gambling showed a better performance than percentage of monthly income lost per gambling episode. Future replications are needed. Nonetheless, using percentage of monthly income lost in gambling as a measurement of monetary losses may lead to more standardized and valid assessments in clinical practice and research on GD.

## Author's contributions

We confirm that all persons designated as authors qualified for authorship. Each author participated sufficiently in the work to take public responsibility for the content. The corresponding author affirms that he had access to all data from the study, both what is reported and what is unreported, and also that he had complete freedom to direct its analysis and its reporting, without influence from the sponsors. The corresponding author also affirms that there was no editorial direction or censorship from the sponsors.

Gustavo C. Medeiros conducted literature searches, the statistical analysis and wrote the first draft of the manuscript. Sarah A. Redden and Samuel R. Chamberlain made edits and provided intellectual input to the first draft of the manuscript. Jon E. Grant designed the study; wrote the protocol; supervised the literature searches and statistical analysis and reviewed the final version of the paper. All authors contributed to and have approved the final manuscript.
